# Students’ performance during practical examination on whole slide images using view path tracking

**DOI:** 10.1186/s13000-014-0208-6

**Published:** 2014-10-30

**Authors:** Slawomir Walkowski, Mikael Lundin, Janusz Szymas, Johan Lundin

**Affiliations:** Poznan University of Technology, M. Sklodowska-Curie Square 5, 60-965 Poznan, Poland; Institute for Molecular Medicine Finland FIMM, University of Helsinki, PO Box 20, FN-00014 Helsinki, Finland; Department of Clinical Pathology, Poznan University of Medical Sciences, Przybyszewski Str. 49, 60-355 Poznan, Poland

**Keywords:** View path tracking, Whole slide image, Practical examination, Viewing behavior, Visualization, Metrics, Digital pathology

## Abstract

**Background:**

Whole slide images (WSIs) used in medical education can provide new insights into how histological slides are viewed by students. We created software infrastructure which tracks viewed WSI areas, used it during a practical exam in oral pathology and analyzed collected data to discover students’ viewing behavior.

**Methods:**

A view path tracking solution, which requires no specialized equipment, has been implemented on a virtual microscopy software platform (WebMicroscope, Fimmic Ltd, Helsinki, Finland). Our method dynamically tracks view paths across the whole WSI area and all zoom levels, while collecting the viewing behavior data centrally from many simultaneous WSI users. We used this approach during the exam to track how all students (N = 88) viewed WSIs (50 per student) when answering exam questions (with no time limit). About 74,000 records with information about subsequently displayed WSI areas were saved in the central database. Gathered data was processed and analyzed in multiple ways. Generated images and animations showed view fields and paths marked on WSI thumbnails, either for a single student or multiple students answering the same question. A set of statistics was designed and implemented to automatically discover certain viewing patterns, especially for multiple students and WSIs. Calculated metrics included average magnification level on which a WSI was displayed, dispersion of view fields, total viewing time, total number of view fields and a measure depicting how much a student was focused on diagnostic areas of a slide.

**Results:**

Generated visualizations allowed us to visually discover some characteristic viewing patterns for selected questions and students. Calculated measures confirmed certain observations and enabled generalization of some findings across many students or WSIs. In most questions selected for the analysis, students answering incorrectly tended to view the slides longer, go through more view fields, which were also more dispersed – all compared to students who answered the questions correctly.

**Conclusions:**

Designed and implemented view path tracking appeared to be a useful method of uncovering how students view WSIs during an exam in oral pathology. Proposed analysis methods, which include visualizations and automatically calculated statistics, were successfully used to discover viewing patterns.

**Virtual slides:**

The virtual slide(s) for this article can be found here: http://www.diagnosticpathology.diagnomx.eu/vs/13000_2014_208

**Electronic supplementary material:**

The online version of this article (doi:10.1186/s13000-014-0208-6) contains supplementary material, which is available to authorized users.

## Background

Whole slide images (WSIs) are digital representations of entire histological glass slides. WSI is a technology which may not only replace the conventional way of viewing slides but also provide new insights into how the slides are examined when making a diagnosis. This aspect is especially interesting in the case of students who take a practical exam in pathology. Tracking viewed WSI areas may explain why a student chose the right or wrong answer. Such knowledge is even more useful when it includes viewing behavior of multiple students for many slides. Creating a tracking infrastructure scalable to many users and analyzing students’ viewing behavior during an exam are the objectives of this paper.

WSIs have been described as a useful and scalable solution for education purposes, including medical education and examination [1]. Digitized histological images can be successfully utilized in web-based learning modules [2]. The WSI system used in this work (WebMicroscope, Fimmic Ltd, Helsinki, Finland) is a tool appreciated by students [3,4]. It has been already utilized in the Poznan University of Medical Sciences in Poznan, Poland since year 2005 to not only enable teaching but also conduct student examination [3,5].

One of the significant advantages of WSIs, compared to traditional histological glass slides, is enabling a wide range of complex analyses which can be performed using digital tools. Specially designed and implemented algorithms can tackle problems like comparing image quality of WSIs acquired using different devices [6] or distinguishing between WSIs containing histopathological patterns characteristic of certain diagnoses automatically [7]. The analytical topics related to WSIs cover the educational area as well. For example, assessment of learning can be extended to investigate how a user interacts with a digital sample. This can include the whole learning process, from analysis of how inexperienced students view educational samples to studies on how experts’ minds work and evaluating pathologists’ performance in less subjective way [8].

One way of recording viewing behavior is eye movement tracking with highly specialized equipment [9], which has also been used to analyze how other medical images are viewed [10]. However, this approach is cumbersome and may be difficult and expensive to adapt for a large number of users. Using a software method is an alternative. It has already been suggested that signals collected from regular WSI viewing hardware, like mouse cursor position [11] and data from slide navigation [12], may have potential in identification of viewing behavior.

We use an innovative view path tracking method, which has been integrated with the WSI software platform WebMicroscope, and is described for the first time in this paper. This method dynamically tracks view paths across the whole WSI area and all zoom levels, centrally collects viewing behavior data from many simultaneous WSI users and requires no specialized equipment.

## Methods

A view path tracking approach was used to create and save information about how a WSI was examined. We tracked and analyzed viewing behavior of students (N = 88) of the Poznan University of Medical Sciences who participated in the practical exam in oral pathology in 2012. This retrospective study is based on routine analysis of the exam results and it is not an experiment. According to the curriculum of the Poznan University of Medical Sciences, this exam is an obligatory form of getting a credit for the oral pathology course. Dental students were informed about the conditions and form of the exam at the beginning of the course. Each student accepted displayed information when logging into the exam system and his or her answer sheet. View path tracking data utilized in the study is anonymous.

Every student was answering 50 exam questions. Each question was in the form of a multiple choice test (with a single correct answer) and had a WSI attached to it. Students spent the vast majority of exam time on viewing these WSIs, while the rest of the time was used by them for reading questions and selecting answers. All students had unlimited time for completing the whole exam. Therefore, the duration of the exam depended on a particular student.

Slides were viewed using regular computer hardware and specially designed WSI viewer software – integrated, but optional part of the current WSI system. Each time a student stopped panning and zooming and image data for the whole view field was loaded, a record was generated and sent to the central database. The record contained coordinates viewed, timestamp and metadata enabling identification of the given student and question. In total, there were almost 74,000 records sent during the exam in 2012. This corresponds to an average of about 750 view fields recorded per student in an exam session or about 15 view fields per WSI per student in an exam session. To enhance the quality of the data, 86 records coming from two students were excluded from the final processing as they were affected by workstation technical issues during the exam. Rest of the data was used for the analysis described in this paper.

Once the exam concluded, the database contained data ready to be processed and analyzed. Two general approaches were used for data analysis: visualization and statistics.

### Visualization

Whole slide images are graphical objects by their nature so illustrating view paths on images results in a human-readable data representation. A single view path is represented by a set of rectangles drawn on top of a slide thumbnail. Each rectangle represents an area which the student displayed on his or her screen while interpreting the slide. To improve clarity, rectangles are filled with semi-transparent color, with opacity depending on a view field’s zoom level, and connected with arrows, to visualize the order of viewing.

**Figure 1:**
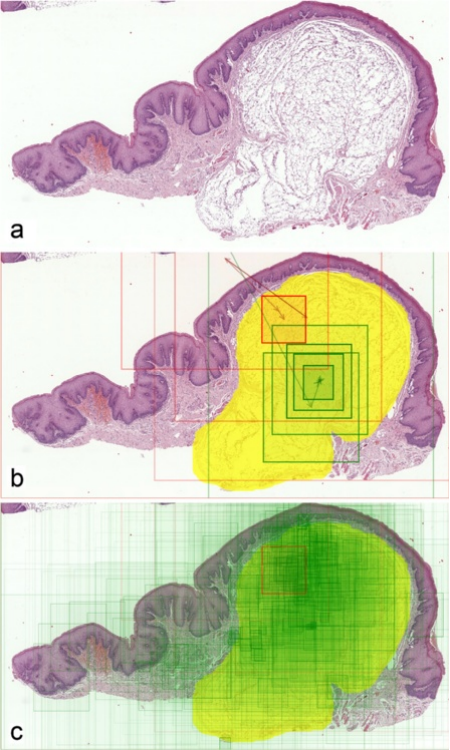
**Visualization process for a question containing a WSI demonstrating submucosal lipoma. (a)** WSI overview (thumbnail). **(b)** Viewing behavior of two students during the exam. Rectangles and arrows denote view paths – green one represents a student who answered the question attached to this WSI correctly, while red one is for a student who gave an incorrect answer. Diagnostic area, which denotes the expected region of interest (ROI), is marked in yellow. **(c)** An aggregated map of areas viewed by all students answering this question; only one student answered incorrectly.

Such an image can be enhanced by marking the regions of interest (ROIs) on which a student should focus to make a diagnosis. Comparing them with the view path location can easily tell us whether a student was viewing an appropriate area of a WSI (Figure 1b).

View paths for the same WSI but coming from multiple students were also compared on a single image. By using two distinctive colors, students answering correctly and incorrectly can be easily distinguished in such visualization. Drawing all students’ view paths on one slide thumbnail provides a map of areas which were most frequently viewed by the whole group (Figure 1c).

**Figure 2:**
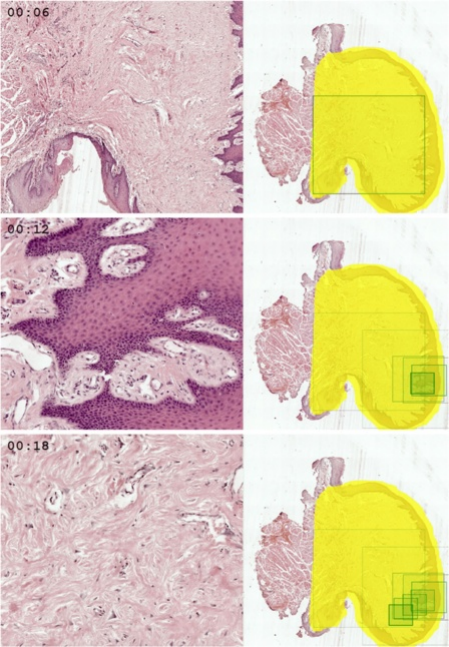
**Three frames from an animation showing how a student viewed a WSI with ‘irritation’ fibroma.** Left side of each frame presents a fragment displayed on student’s screen at the given moment, while right side shows a WSI thumbnail with positions of the current and previous view fields marked as green rectangles.

Having a timestamp associated with each database record enables creation of not only static images but also animations. In this case, rectangles representing single view fields appear at the same pace as a student was panning and zooming to corresponding regions. This method illustrates time spent on viewing particular areas. These areas can be simultaneously displayed next to the slide overview, forming a dynamic replay of what student was seeing on his or her screen (Figure 2, Additional file 1). Naturally, the speed of the animation can be adjusted and view paths from multiple students can be animated on a single slide overview (Additional file 2).

### Measures

Analysis of visualizations by a human is not scalable to discover viewing behavior of many students interpreting many WSIs. Such visual assessment may also not be precise enough to draw general conclusions. To address these problems, a set of 26 statistics was designed and calculated to numerically describe viewing behavior. This approach enables aggregation of records for all view fields in a view path into a single set of metrics. Once calculated for each student and each question answered, the metrics may be further compared and aggregated across students and slides.

Calculated statistics included both simple metrics, like total viewing time and number of view steps, and more advanced formulas, describing spatial relations between view fields. The latter included the following measures, selected for further analysis:

- ‘Regions of interest (ROIs) viewed’, depicting how much a student was focused on diagnostic areas of the slide. This measure was calculated as a percentage of view field area which included an ROI area and aggregated across all view fields except for the first one, which was by default presented to a student right after opening the WSI and does not represent student’s navigation. The aggregation was done using an arithmetic mean weighted by the time each view field was displayed on a screen.
- Average zoom level, also weighted by view fields’ viewing time, used to describe at what level of detail a student was interpreting the slide.
- Dispersion, describing how scattered or concentrated the viewed areas were. This concept was expressed as an average distance between each pair of view fields. Similarly to ‘ROIs viewed’ measure, the first (default) view field displayed after opening the WSI was not included in the calculation.

In this paper, we focused on comparing the behavior of students answering a given question correctly and students whose answers were incorrect. Measures mentioned above were chosen based on how well they differentiate these two groups of students, according to initial tests and analysis.

## Results

Selected metrics were used together with visualizations to compare students’ viewing patterns within the same questions. Then, an aggregation of measures across multiple questions allowed us to make a broader comparison and draw more general conclusions.

**Figure 3:**
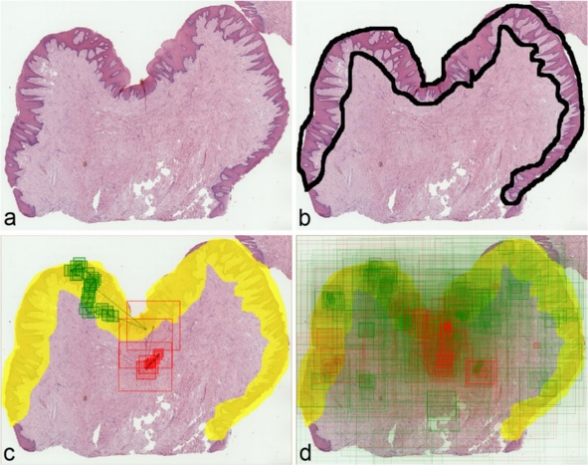
**Visualization steps for a question containing a WSI with benign reactive keratosis. (a)** Slide overview. **(b)** Region of interest (diagnostic area) marked by a pathologist for analysis purposes. **(c)** View paths for two answers (correct = green and incorrect = red), coming from two students. **(d)** Aggregated map of view paths from all students’ answers for this question.

**Figure 4:**
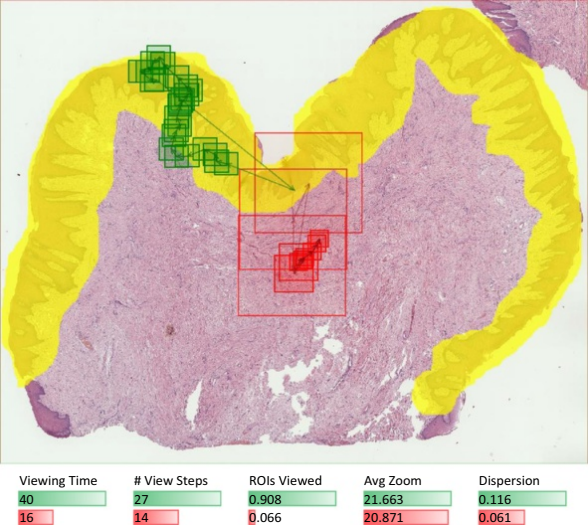
**Comparison of two students interpreting a WSI with benign reactive keratosis.** Visualization and calculated measures show that in this case, the student who answered correctly (green rectangles) was more focused on the diagnostic area (yellow) and went through more view fields, which were also more dispersed across the slide – all compared to the student who gave a wrong answer (red rectangles).

We compared viewing behavior of two students interpreting a WSI with benign reactive keratosis (Figure 3). Based on visual assessment, we can clearly see that the student who answered correctly was much more focused on diagnostic areas, looked at more single fields of view, which were more dispersed across the WSI – as compared to the student who gave a wrong answer (Figure 4). These three observations are confirmed with the measures calculated for these students’ view paths. Another measure, which also differentiates these answers, is total viewing time – it is longer for the examinee who answered correctly.

Such conclusions are specific to these two students viewing this particular WSI. In fact, they are very different from more general findings presented below. This shows the complexity of analyzing viewing behavior of many students interpreting many WSIs. However, the set of methods presented in this paper makes the analysis possible on multiple aggregation levels.

**Figure 5:**
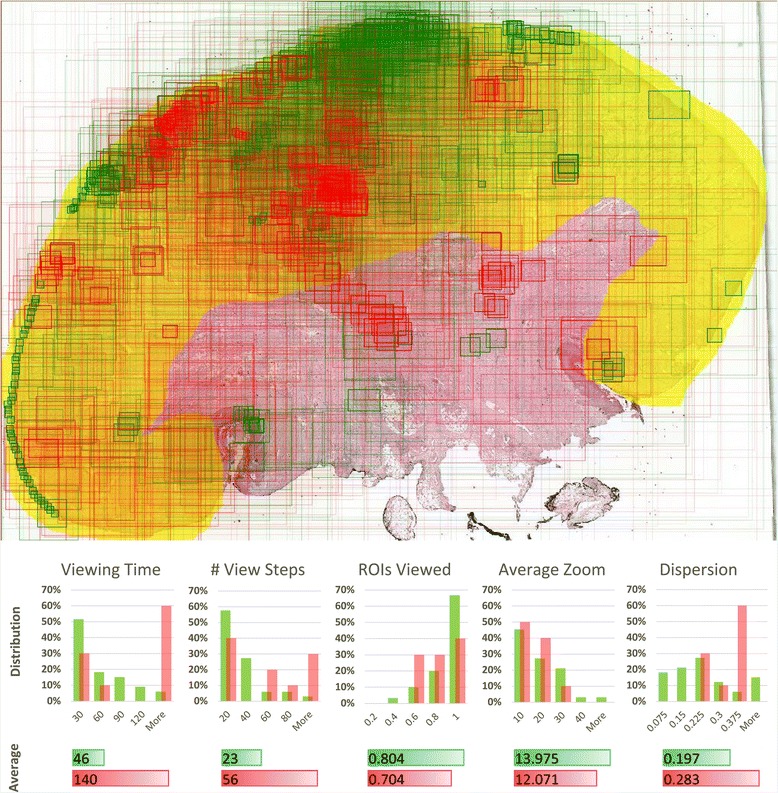
**View paths and metrics for all students interpreting this WSI (squamous cell carcinoma).** Statistics include distributions with average values of measures calculated for each student. Numbers show that for this question, students who gave incorrect answers (indicated with red) used more time to view the slide and went through more view fields. Other metrics suggest that students answering correctly (indicated with green) were a bit more focused on the diagnostic area, used higher magnification and the areas they viewed were less dispersed across the slide.

In case of a question containing a slide with squamous cell carcinoma, we compared view fields for all students’ answers at the same time (43 answers with 1,330 view steps in total). Visualization of view fields coming from all students gives some idea about slide areas on which most students were focused (Figure 5). However, it would be difficult to draw any conclusion about differences in behavior of students answering correctly and incorrectly if based solely on such an image. Comparing measures calculated from the raw viewing data helps in this task. Average values and distributions were calculated for each measure within groups that gave correct and wrong answers, respectively. It can be seen that for this question, students answering incorrectly tend to view the slide longer, going through more view fields. Differences in average values and distributions of other metrics are smaller but it can be still noticed that students with correct answers are generally a bit more focused on ROIs, use higher magnification and move less across different areas of the slide.

Finally, metrics were calculated and compared across multiple questions and their corresponding WSIs used in the exam. Because we were comparing behavior of students answering correctly and incorrectly, questions with at least 5 correct and 5 incorrect answers were selected. This resulted in a set of 26 questions with 1,033 students’ answers and 19,996 view fields in their view paths on corresponding slides. Ten of these questions had ROIs marked on WSI overviews for the analysis purposes, which made calculating ‘ROIs viewed’ measure possible for them.

**Figure 6:**
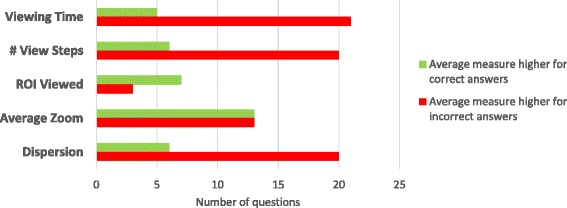
**Comparison of numbers of questions with average measure value higher for correct or incorrect answers.** The set of questions for this analysis was limited to 26 questions with at least 5 correct and 5 incorrect answers. In most questions, average viewing time, number of view fields and dispersion are higher for students answering incorrectly. Students with correct answers seem to focus on diagnostic areas more often.

As characteristics of each slide may be different, aggregating absolute metrics’ values across multiple WSIs may be biased. Instead, questions with average value higher for correct answers and questions with average value higher for incorrect answers were just counted for each measure. This resulted in overall statistics shown in the chart in Figure 6. Conclusions which can be drawn from them are close to the outcome of the squamous cell carcinoma slide analysis above. In most questions, when students give wrong answers, they tend to spend more time on interpreting the slide and go through more view fields, which are also more dispersed than for students answering correctly. The latter ones seem to be more focused on appropriate ROIs. Magnification level does not appear to be correlated with answer correctness in this overall comparison.

## Discussion

The results show that the view path tracking method can be successfully used to get information about how students viewed WSIs during a practical exam in oral pathology. Obtained raw data was processed, analyzed and presented in various ways, on different aggregation levels. This resulted in multiple conclusions about students’ viewing behavior during the exam.

The software-based tracking method for WSIs appeared to be a good choice for the exam session environment. This approach has certain advantages over solutions described in [9,11], which involve eye movement tracking. Once implemented on a WSI software platform, our method is easy to adapt and requires no changes in the way how WSIs are normally viewed. Compared with the approach presented in [12], it utilizes standard and more user-friendly graphical interface, scales well for multiple simultaneous users and collects tracking data centrally.

The method works well because of typical navigation (panning and zooming) required when interpreting a WSI. On the other hand, our approach cannot determine which part of the currently displayed fragment is being actually viewed. This is the task which can be accomplished using eye movement analysis and adding it to the navigational tracking could provide even more detailed results. However, scalability and adaptability of the current solution would be degraded because of extra equipment and preparation required by the eye movement tracking.

Application of the approach described in this paper can go beyond drawing conclusions about WSI viewing behavior among students. It would be interesting to enable the tracking method in WSI viewers used by a group of expert pathologists for longer time. In this way, we could get a large set of data, which could help us better understand the process of making diagnoses by pathologists or evaluate their performance in a better way, like suggested in [8]. Moreover, WSI tracking data coming from experts could be used by computer-aided diagnosis algorithms as training signals, which could help in automatic identification of important diagnostic areas in the slides containing certain diseases.

In the education area, one can imagine that information from view path tracking could be utilized as an element of student’s performance assessment. For example, a student who efficiently viewed a WSI and gave the correct answer could obtain a higher score than a student who also answered correctly but his or her way of viewing the slide suggested that he or she was not really prepared for the question. Furthermore, extending similar tracking approaches to the learning process can play an important role in the future of digital education. Discovering the behavior of students when they learn and acquire knowledge using digital technologies can help in making the courses more efficient. Collecting and automatically analyzing detailed signals about users’ behavior in real time can lead to developing immersive educational applications, which provide fast and direct feedback to the students. Because of scalability of such solutions, they could be applied to both regular classes and online courses.

## Conclusions

It can be concluded that view path tracking appeared to be a useful method of discovering viewing behavior of a large group of users interpreting many whole slide images. Although no specialized hardware was used, gathered data was a valuable resource. It was visualized in multiple ways, providing many useful insights into both individual viewing behaviors and patterns occurring across multiple students. Automatically calculated statistics reflected some viewing characteristics, confirmed visual observations and enabled generalization of some findings for multiple students and exam questions.

## Additional files

Additional file 1:
**Animation presenting a view path for a student interpreting a WSI with ‘irritation’ fibroma.** Left part of the animation represents the area which was displayed on student’s screen at the time indicated in the upper left corner. Time is measured starting from the moment when the first view field was loaded after opening the WSI by the student. Right side shows the WSI overview, which includes diagnostic area (in yellow) and cumulative visualization of view fields subsequently displayed by the student (green rectangles). Animation speed is 2× compared to real time. Additional file 2:
**Animation showing view paths for all students interpreting a WSI with cervical lymphoepithelial cyst.** View fields subsequently displayed by all students answering a question attached to this WSI are visualized on a single WSI overview. Students answering correctly are differentiated from students answering incorrectly by using green and red rectangles, respectively. Animation of multiple view paths is synchronized by using the time of the first view field in each view path as the common animation start time. Fragments viewed at the given moment are distinguished by bold borders. Animation speed is 5× compared to real time.

## Abbreviations

WSI: Whole slide image
ROI: Region of interest
