# Supplementary figures and images for: Students’ performance during practical examination on whole slide images using view path tracking
Source: Diagn Pathol. 2014 Oct 30;9:208. doi: 10.1186/s13000-014-0208-6 (PMC4251864; doi:10.1186/s13000-014-0208-6)

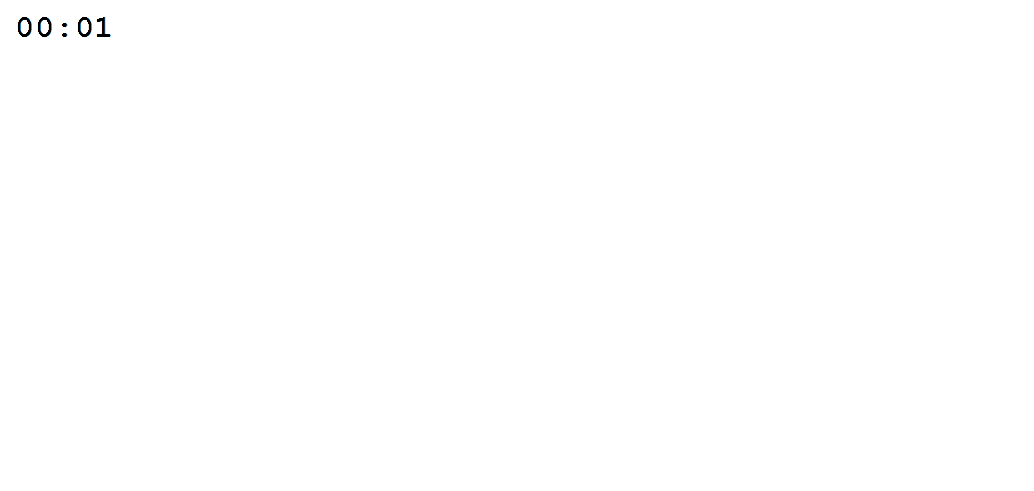

Supplement: Additional file 1: — Animation presenting a view path for a student interpreting a WSI with ‘irritation’ fibroma. Left part of the animation represents the area which was displayed on student’s screen at the time indicated in the upper left corner. Time is measured starting from the moment when the first view field was loaded after opening the WSI by the student. Right side shows the WSI overview, which includes diagnostic area (in yellow) and cumulative visualization of view fields subsequently displayed by the student (green rectangles). Animation speed is 2× compared to real time. [file 13000_2014_208_MOESM1_ESM.gif]

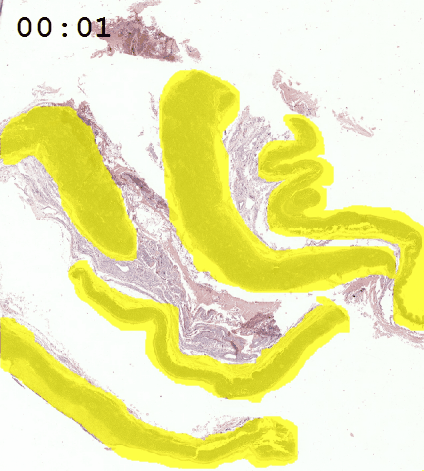

Supplement: Additional file 2: — Animation showing view paths for all students interpreting a WSI with cervical lymphoepithelial cyst. View fields subsequently displayed by all students answering a question attached to this WSI are visualized on a single WSI overview. Students answering correctly are differentiated from students answering incorrectly by using green and red rectangles, respectively. Animation of multiple view paths is synchronized by using the time of the first view field in each view path as the common animation start time. Fragments viewed at the given moment are distinguished by bold borders. Animation speed is 5× compared to real time. [file 13000_2014_208_MOESM2_ESM.gif]
